# Epithelial Growth Factor-Anchored on Polycaprolactone/6-deoxy-6-amino-*β*-cyclodextrin Nanofibers: In Vitro and In Vivo Evaluation

**DOI:** 10.3390/polym13081303

**Published:** 2021-04-16

**Authors:** Edgar D. Moyers-Montoya, René Gerardo Escobedo-González, Claudia L. Vargas-Requena, Perla Elvia Garcia-Casillas, Carlos A. Martínez-Pérez

**Affiliations:** 1 Institute of Engineering and Technology, Autonomous University of the City of Juárez, UACJ ve. Del Charro 450 Norte, Ciudad Juárez 32310, Mexico; edgar.moyers@gmail.com (E.D.M.-M.); pegarcia@uacj.mx (P.E.G.-C.); 2 Department of Industrial Maintenance, Technological University of the City of Juárez, Av. Universidad Tecnológica No. 3051, Col. Lote Bravo II, Ciudad Juárez 32695, Mexico; renegerardo.escobedo@gmail.com; 3 Institute of Biomedical Sciences, Autonomous University of the City of Juarez, UACJ, Henry Dunant #4600, Ciudad Juárez 32310, Mexico; cvargas@uacj.mx

**Keywords:** nanofibers, wound healing, polycaprolactone, cyclodextrin, EGF

## Abstract

Polycaprolactone (PCL) is a well-known FDA approved biomaterial for tissue engineering. However, its hydrophobic properties limit its use for skin wound healing which makes its functionalization necessary. In this work, we present the fabrication and evaluation of PCL nanofibers by the electrospinning technique, as well as PCL functionalized with 6-deoxy-6-amino-β-cyclodextrin (aminated nanofibers). Afterwards, epithelial growth factor (EGF) was anchored onto hydrophilic PCL/deoxy-6-amino-β-cyclodextrin. The characterization of the three electrospun fibers was made by means of field emission scanning electron microscopy (FESEM), Fourier transform infrared spectroscopy-attenuated total reflectance (FTIR-ATR); Confocal-Raman Spectroscopy were used for elucidated the chemical structure, the hydrophilicity was determined by Contact Angle (CA). In vitro cell proliferation test was made by seeding embryonic fibroblast cell line (3T3) onto the electrospun mats and in vivo studies in a murine model were conducted to prove its effectivity as skin wound healing material. The in vitro studies showed that aminated nanofibers without and with EGF had 100 and 150% more cell proliferation of 3T3 cells against the PCL alone, respectively. In vivo results showed that skin wound healing in a murine model was accelerated by the incorporation of the EGF. In addition, the EGF had favorable effects in epidermal cell proliferation. The study demonstrates that a protein of high biological interest like EGF can be attached covalently to the surface of a synthetic material enriched with amino groups. This kind of biomaterial has a great potential for applications in skin regeneration and wound healing.

## 1. Introduction

Nanofiber polymer scaffolds prepared by electrospinning techniques are being used for different therapies and treatments due to the capacity of these kind of materials to emulate the morphology of the extracellular matrix [1,2]. It is already well known that electrospinning is an efficient and economical technique that allows the fabrication of polymer nanofiber [3]. There are several biocompatible and biodegradable polymers that have shown great performance for scaffolds manufacturing like Poly-l-Lactic Acid (PLLA), Poly (glycolic acid) (PGA), Polycaprolactone (PCL), and blends of them [4,5,6]. All of them are non-toxic and approved by the U.S. Food and Drug Administration (FDA) for clinical use in humans. PGA has been blended with Hydroxyapatite/PLLA to accelerate degradation in order to obtain a better performance in the bioactivity and osteoconductivity cause by the improvement of hydrophilicity by the incorporation of PGA [7]. On the other hand, PCL has unique mechanical properties which make it of easy processability and morphology control in the electrospinning process. PCL mimic the extracellular matrix; in addition, it is biocompatible and bioresorbable making it an excellent candidate for wound healing dressing. However, PCL has a lack of chemical interaction sites to anchor molecules of biological importance. Therefore, PCL for tissue regeneration proposals requires some strategies to improve its interaction capacity. Commonly, cell scaffolds are enriched with biomolecules, in a way that regeneration reaction in the tissues could be potentiated [8,9,10].

Some strategies which search a synergistic effect on their properties include the addition of biological interest molecules by mixing different materials with or without covalent bonds [9,10,11,12]. Further, the preparation of inclusion complexes with amphipathic cyclic molecules by molecular noncovalent interactions has been used in the preparation of biomaterials. One example are the cyclodextrins, which allow non-covalent bonds with hydrophobic molecules. Since they are rings that have a hydrophobic center and expose outwardly hydroxyl groups that provide hydrophilic interaction. A complex is formed where hydrophobic molecule as PCL is immersed inside of cyclodextrins structure by the driving force of hydrophobic interactions, in this way, cyclodextrins provide to PCL a great number of hydroxyl groups with potential covalent binding with certain biomolecules [13].

After an injury, several intracellular and intercellular processes should be activated and coordinated to restore tissue integrity and homeostasis where a long list of regulatory biomolecules are involved. Peptide growth factors are key molecules in re-epithelialization, the epithelial growth factor (EGF) family regulates re-epithelialization and wound repair. EGF has been encapsulated in different kind of polymers and then applied directly around the lesion to deliver it. However, EGF tends to a burst release with the need for search new strategies and systems that would incorporate and release EGF with good results in skin wound healing [14].

In this context, we present the physicochemical characterization, as well the in vitro and in vivo wound repair evaluation of the electrospun nanofibers made with inclusion complex between PCL and 6-deoxy-6-amino-β-cyclodextrin (aminated nanofibers) which was incorporated in order to provide sites to anchor the EGF, this will reinforce the evaluation reported in our previous work [13]. This material was designed to increase the biocompatibility and bioactivity of the PCL by the addition of amine groups on the surface to provide high hydrophilicity and the possibility of covalently attached EGF molecules by the interaction through the primary amines. The results show that this system stimulates cell proliferation and wound healing when was applied to heal a wound in a murine model.

## 2. Materials and Methods

### 2.1. EGF Immobilization on Surface of Aminated Nanofiber

The aminated nanofibers were prepared in accordance with a previous work [13]. The used bulk material of PCL with *β*-CD-NH_2_ was obtained in agreement with the procedures of Yoshinori Kawaguchi et al. [15] and Jiahan Zhan et al. [16]. Poly(e-caprolactone) PCL with a MW of 70,000–90,000 Da, Glutaralodehyde solution grade II, 25% in H2O, Epidermal Growth Factor from murine submaxillary gland were acquired from Sigma-Aldrich (St. Louis, MO, USA); Acetone, 2,2,2-Trifluoroethanol (TFE), dimethylformamide (DMF); All of them were purchased from JT Baker (Ecatepec, México State, México), and used without further purification.

A PCLwas used to prepare a solution with acetone at concentration of 1.66% (*w/v*). The solution was heated in an oil bath until 50 °C. Once the temperature was reached, a solution of *β*-CD-NH_2_ in DMF with concentration of 0.833% (*w/v*) was added dropwise and stirred for 2 h. Then the mixture was let to cool down at room temperature and the acetone was evaporated overnight under slowly stirred. The uncomplexed *β*-CD-NH_2_ (6-deoxy-6-amino-*β*-cyclodextrin) was removed by washing the mixture several times, then it was dried at 37 °C under reduced pressure of 25 in Hg. Afterwards, the PCL with *β*-CD-NH_2_ was electrospun [13].

The amine groups in the nanofibers were quantified using the ninhydrin test [17]. First, a calibration curve using L-valine was made using an L-valine standard solution with concentration of 1.01 × 10^−3^ M in water and its diluted solution at 1.03 × 10^−5^ M. The ninhydrin solution used for quantification was prepared in freshly and darkness prepared ninhydrin ethanolic solution for all tests, the concentration used was 1.97 × 10^−3^ M. For a typical assay, the calibration curve values were obtained mixing volumes of 0 to 5 mL of L-valine standard solution with 1 mL of the ninhydrin solution and diluted in absolute ethanol to the final volume of 6 mL. The prepared mixes were introduced in a test tube, sealed by a bung and a parafilm and heated at 100 °C and stirred vigorously for 90 min followed by a cool-down to room temperature. UV-Vis absorption spectra of the solutions were recorded between 400 and 700 nm using Thermoscientific nanodrop 2000. The amine groups on nanofibers were measured with the same procedure, using 1 cm^2^ per sample and all the measures were determined by triplicated [18].

The epithelial growth factor was covalently attached following an immobilization technique, using glutaraldehyde on amine groups of nanofibers [19,20]. Initially, the electrospun nanofibers were washed with a PBS solution, then the mats were immersed in 25% (*v/v*) glutaraldehyde solution to activate the surface by the conjugation of glutaraldehyde with the amino groups of the material, it was put in a shaker for 1 h at room temperature. Afterwards, it was washed with phosphate solution to remove the unattached glutaraldehyde, then, it was dipped overnight into an EGF solution (10 µg/mL) at 4 °C under stirring. The final concentration was determined by UV-Vis spectroscopy and the grafted protein-material was quantified by absorbance difference between the initial contact protein concentration and the final protein concentration [21]. Aminated nanofiber with EGF were washed with PBS and dried at room temperature in a vacuum chamber [22,23,24,25].

### 2.2. Aminated Nanofibers with/without EGF Characterization

The characterization of the electrospun materials was made by means of Field Emission Scanning Electron Microscopy (JEOL JSM 7000F, JEOL, Tokyo, Japan). The EGF distribution on the nanofibers was observed by means of Confocal Raman Microscopy (Alpha 300 AR, WiTec GmbH, Ulm, Germany) with an Olympus optical setup and 542 nm laser, a spectral scan was carried out allowing to create an image with the discrimination of the representative signals, as well as a composite image that allows to see the distribution of both signals [26,27]. Further, the obtained nanofibers were characterized by Fourier transform infrared with attenuated total reflection FTIR-ATR (Nicolet 6700 ThermoScientific) from 400 to 4000 cm^−1^ range with a resolution of 4 cm^−1^, 32 repetitive scans were automatically added for each measurement Ge crystal was used for the ATR mode. The wettability of the materials was evaluated by measuring in Drop Shape Analizer-DSA30 (Krüss) using a physiological solution.

### 2.3. In Vitro Biocompatibility Study (3T3 Cell Culture)

Biocompatibility of “Aminated nanofibers with/without EGF” was measured using the methodology of MTT assays [28]. The mouse embryonic fibroblast cell line (3T3) was obtained from ATTC and cultured in Dulbecco’s modified Eagle’s medium (DMEM). The culture medium enriched with 10% of fetal bovine serum was put in an incubator at 37 °C and atmosphere of 5% CO_2_. Moreover, to prevent contamination penicillin/streptomycin (1%) was added, the culture media were changed every 48 h. After rinsing with a PBS it was incubated with a 0.05% trypsin-EDTA solution. The activity of the trypsin was terminated after 1 to 2 min by the addition of 5 mL of complete growth medium. The mixture was centrifuge for a few minutes and the supernatant discarded. The cells were suspended in a fresh medium and then seeded into culture flasks for further propagation and subsequent passages. Cells from 2nd to 4th passages were seeded on the nanofibers with and without EGF in order to analyze their growth and proliferation for 14 days. Cell viability was measured according to Mosmann [28] using MTT reagent dissolved in PBS (0.5 mg/mL). After 24 h, the culture medium was replaced by a fresh medium of DMEM and 10% FBS with diluted MTT, then incubated at 37 °C in a CO_2_ incubator for 1 h. Afterwards, formazan crystals were dissolved in 100 μL of DMSO. The reduction of MTT was quantified using a microplate reader (Bio rad, Hercules, CA, USA) by measuring the absorbance at 570 nm [29]. Viability was calculated as shown in Equation (1):

(1) $$Viability\;\left(\%\right)=\frac{B}{A}\times 100\;$$

where *A* is the absorbance value of the control group and *B* is the absorbance value of nanofiber tested.

### 2.4. T3 Cell Proliferation in Contact with the Scaffold

Cell proliferation was measured by place, in a 12-well plate, round pieces of the material with a diameter of 1.5 cm previously disinfected with UV light for 30 min. As control, it was used al tissue culture plate. 3000 cells per well were seeded for and incubated at 37 °C in a CO_2_ incubator for a period of 3, 7, 10, and 14 days. The cells were rinsed with PBS and incubated with 0.05% trypsin-EDTA at 37 °C in a humidified atmosphere with 5% CO_2_. After 1 to 2 min the activity of the trypsin enzyme was terminated by the addition of complete growth medium and then centrifuged for 5 min at 3000 rpm and the supernatant threw-away. The cells were suspended in 1 mL of fresh medium followed by taking 20 μL of it and mixed with 20 μL of Trypan blue; 10 μL of the mix were placed in Neubauer camera and counted in the 4 quadrants. The total number of cells in 1 mL was calculate by the following formula [30,31]

(2) $$proliferation=\frac{N\times FD\times 10000}{4}$$

where *N*, *FD*, 10,000, and 4 are value cells in the fourth regions, dilution factor and correction factor, and the observed region number, respectively. Statistical analysis of these results was executed by the PRISMA statistical program.

### 2.5. In Vivo Study: Wound Healing Test

In vivo evaluation was made using a Murine model. The murine specimens were anesthetized with isoflurane and the dorsal area was shaved. Circular wounds of 1 cm in diameter were produced using a sterile skin biopsy punch, the wounds were digitally photographed. Then, before to be applied the materials (aminated nanofibers with and without EGF) were sterilized for 30 min in a UV lamp. The animals were taken care of and monitored for 14 days receiving a regular diet [32,33,34,35]. The closing diameter wounds were measured and photographed every day. Wound areas were measured by IMAQ vision of National Instruments Corporation [36,37,38]. All the treatments were made by triplicate, and the statistical analysis of wounds healing percentage was carried out by the PRISMA statistical program. All experimental procedures were approved by the UACJ biomedical Science Institute Ethics Committee (Approval date: January 2017) according to the National Legislation of the use of animals for (NOM-062-ZOO-1999) and the National Institutes of Health (NIH) Guide for the Care and Use of laboratory animals.

## 3. Results and Discussion

The grafted electrospun materials with proteins have been reported by the glyceraldehyde junk reaction, using as base to graft aminated surfaces. In this sense, the amine groups on the surface of the aminated nanofibers were quantified in order to determine the capacity to attach the epithelial growth factor. The quantification was made by ninhydrin method [17] using the L-valine amino acid as standard for the calibration curve. The material absorbance was converted in amine concentration and consequently to amine groups per sample, the analysis showed an average content of 1.13 × 10^17^ amine groups per cm^2^. The number of amine groups on the surface of the aminated nanofibers was in the range of the obtained values by other works [39]. The high value of amine groups on the nanofibers surface suitable for protein immobilization.

### 3.1. EGF Immobilization on Surface of Aminated Nanofiber Scaffolds and Characterization

**Figure 1:**
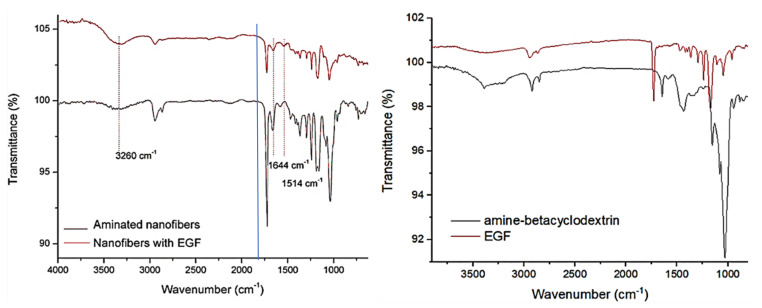
Fourier transform infrared with attenuated total reflection (FTIR) spectra of electrospun materials with and without attached epithelial growth factor (EGF).

The epithelial growth factor (EGF) was anchored onto the aminated nanofibers mats by the immersion of the fibers in an EGF solution. The grafted protein was quantified by absorbance difference between the initial contact protein concentration and the final protein concentration. An average grafted value of 0.02 mg per cm^2^ was obtained, this value corresponds to 2.67 × 10^14^ protein molecules grafted on the surface. The spectroscopic characterization of the materials worked was made by infrared spectroscopy. In Figure 1 the IR spectra measured from 4000 to 400 cm^−1^ for the started materials, inclusion complex before and after the attachment of the EGF is shown.

Infrared spectrum from starter materials revealed similar bands, the both compounds, aminocyclodextrin and EGF showed broad band around 3500 cm^−1^ corresponding at amine-amide and hydroxyl groups, also CH2 stretching bands (2900–2800 cm^−1^); however, in aminocyclodextrin the amine group bending band is observed around 1590 cm^−1^, meanwhile the amide bands were observed to less wavenumber and amide carbonyl group and carboxyl group are showed at 1700 cm^−1^ [13]. The aminated nanofibers with and without EGF showed a distinctive absorption band at 1724 cm^−1^ due to the abundant carbonyl groups in the structure of the PCL, also the C-O and C-C bonds stretching band (1240 cm^−1^) of PCL chain are exhibited. In the structure with EGF the IR spectra showed the characteristic bands of amide groups at 1644 and 1514 cm^−1^, which are attributed to the grafted protein in the material indicating the presence of the EGF in the material [39,40,41]. The band around 1644 cm^−1^ is assigned to both a random coil and α-helix conformation of the protein, since the both materials contains analogous nitrogenated groups (amine and amide), related IR bands were observed from these groups in same wavenumber range (1590–1510 cm^−1^). However, it has been reported the vibrational mode of amide II from in-plane N-H bending appeared at lowest wavenumber than the primary amine N-H bending. In this case, it corresponds to the primary amine group of aminocyclodextrin [42]. The two materials exhibited widen bands attributed to hydroxyl and amine groups of aminocyclodextrin for aminated nanofibers and the aminocyclodextrin and EGF for aminated nanofibers with EGF. Additionally, these bands showed more resolution and with a shift in the amine and hydroxyl band that is attributed to the change in hydrogen bond capability when the EGF is attached. Furthermore, the band of hydrogen in hydro carbonated chains asymetrical and symetrical stretching (2800 cm^−1^) increases its intensity due to the EGF attached. These similitudes on the IR signals, Raman Spectroscopy was used to complement the IR results and let us confirm the presence of EGF anchored by amide bond.

**Figure 2:**
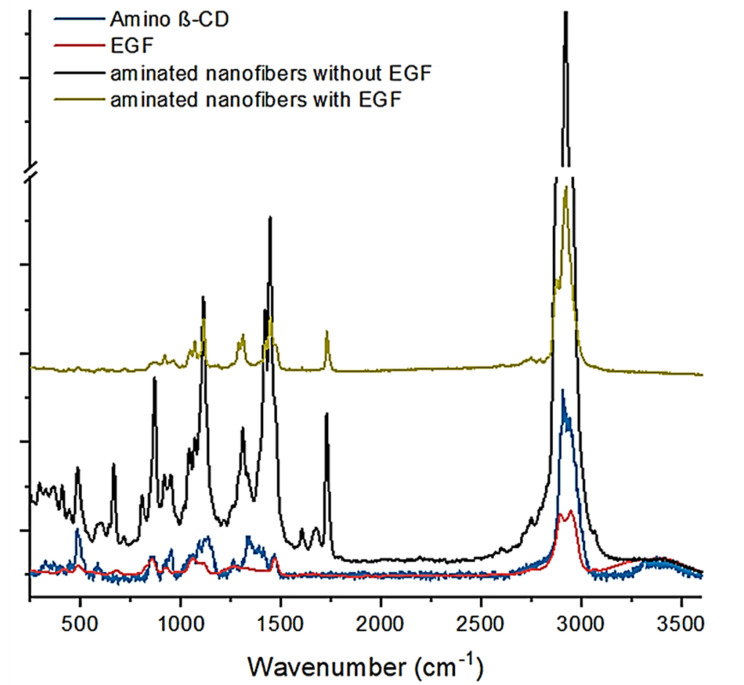
Raman spectra of started materials and the nanofibers with and without EGF.

The vibrational spectroscopic characterization of aminated nanofibers was continued by Raman spectroscopy. The results (Figure 2) showed in the aminated nanofibers with EGF the presence of the amide III band (1316–1285 cm^−1^) attributed to the peptide bonds of proteins. However, the aminated nanofibers without EGF did not show this band [43]. In addition, one band at 2880 cm^−1^ is displayed in the nanofibers with EGF and the same band is exhibited by EGF pure molecule spectra. Moreover, the band assigned to amine group (1606 cm^−1^) in aminated nanofibers without EGF [13] decreased its intensity with the presence of the EGF, this is attributed as consequence of the protein attachment in amine groups of the aminated nanofibers.

**Figure 3:**
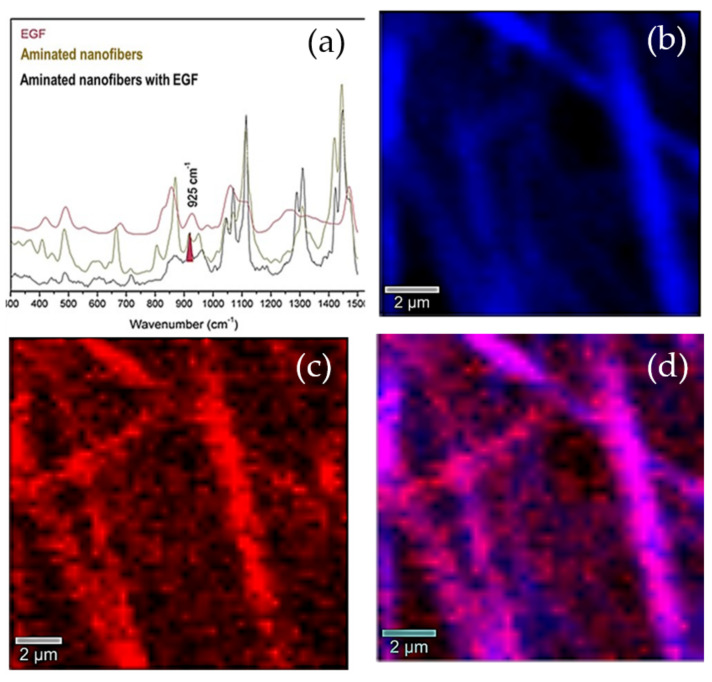
Raman spectra and label free 2D images of aminated nanofibers with EGF. (**a**) selected Raman spectra bands (**b**) The blue image is generated by the integration of signal at ~2857 cm^−1^ attributed to the C-H stretching vibrations in polycaprolactone (PCL). (**c**) The image in red is calculated by the integration for a center of mass of the peak at ~925 cm^−1^ attributed to the EGF. (**d**) The image in purple represents the distribution of EGF in the aminated nanofibers with EGF.

Complementarily, the aminated nanofibers with EGF were studied by confocal Raman spectroscopy (CRS) (Figure 3). CRS collected full Raman spectra sequentially from each location, generating a colored map based on one characteristic Raman band. The CRS images for aminated nanofibers with EGF were generated and coupled to the charge-coupled device (CCD) of the camera to visualize an image-map of material constituents where the selected signal appears during the mapping generating a compositional visual description. Figure 3 shows the structural components of the evaluated material: here the signal band of aminated nanofibers at 925 cm^−1^ (highlighted in red) is attributed to the EGF, the CH_2_ stretching band at 2857 cm^−1^ (highlighted in blue) highlighted to generate a complete image of the nanofibers which allowed us to correlate the position of the EGF across the nanofibers. The EGF distribution in the nanofibers is observed when both CRS images are spliced, causing the appearance of a magenta color due to the combination of red and blue. These images evidence that amine groups and in consequence the EGF are both well distributed along the PCL fibers.

**Figure 4:**
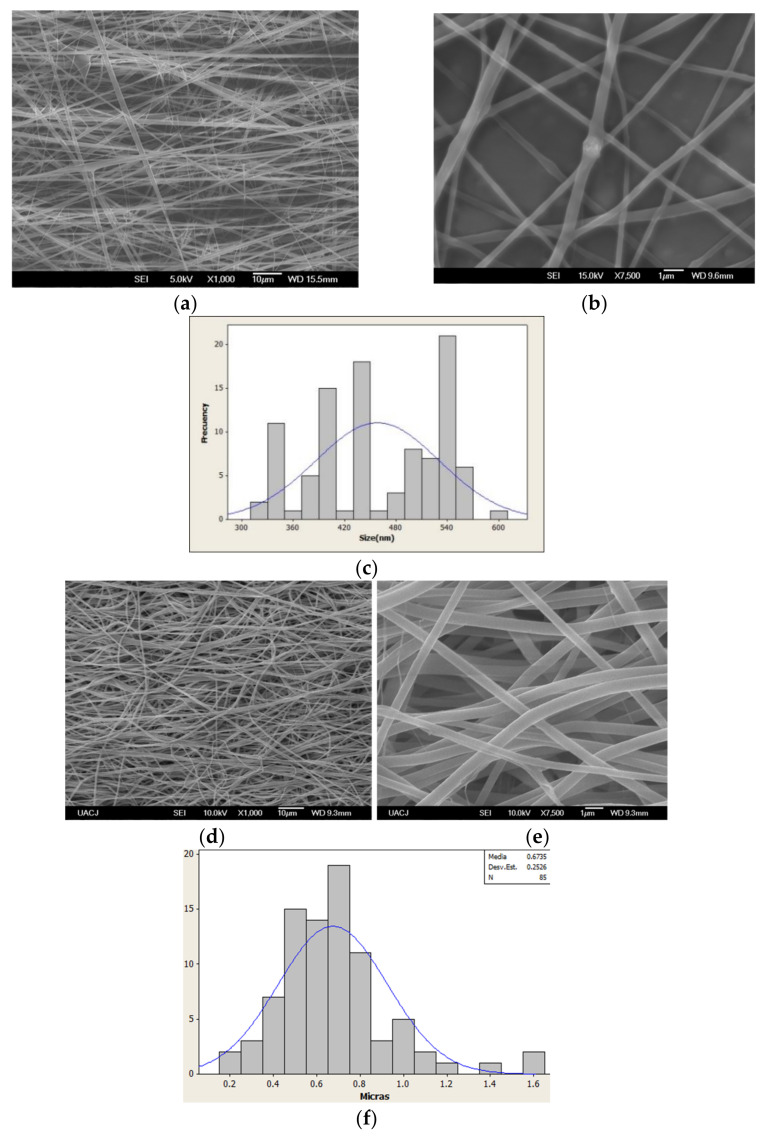
Aminated nanofibers without EGF: (**a**) 1000× (**b**) 7500× (**c**) nanofiber diameter distribution; with EGF: (**d**) 1000× (**e**) 7500× (**f**) nanofiber diameter distribution.

Figure 4 shows scanning electron microscopy (SEM) pictures where the fibrillar structure of the mats can be appreciated, the aminated nanofibers with EGF were thick due to the EGF grafting in comparison with the aminated nanofibers without EGF. The nanofibers with EGF have diameter was mainly in the range of 400 to 800 nm, being that slightly wider than the nanofibers without EGF (average size to 462 ± 77 nm). However, there was no change in the structure and morphology of the aminated nanofibers with EGF and without EGF, but a decrease in the fiber alignment was observed for the aminated nanofibers with EGF. This change could be explained by the exposure to protein’s aqueous dissolution. It has been proposed that the fibers would increase the porosity and distance between them when the fibers are exposed to water, in consequence it results in fibers misalignment [44,45,46].

**Figure 5:**
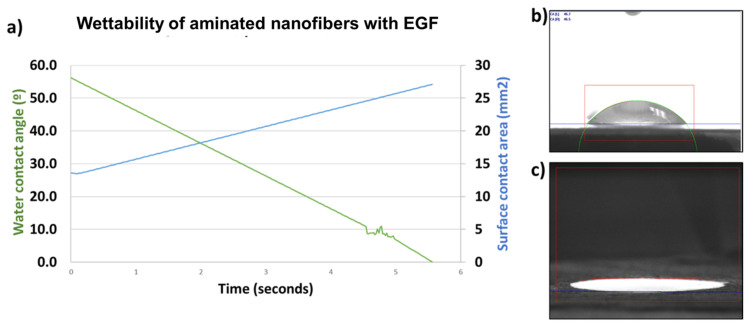
Wettability of aminated nanofibers with EGF. (**a**) water contact angle (°) and surface contact area (mm^2^) vs. time; (**b**) contact angle image (Kruss instrument on time 0 s start measurement); (**c**) contact angle image (Kruss instrument on time 5.56 s and measurement, the image is shown in color contrast to be able to observe the area generated by the drop).

In Figure 5, the wettability of the aminated nanofibers with EGF is compared with the aminated nanofibers. It can verify that the wettability of the material was not compromised with the EGF anchoring treatment. As expected, the anchoring of EGF a hydrophilic polypeptide maintained its high hydrophilicity according to statically measure of the contact angle for aminated nanofibers and aminated nanofibers with EGF being 1.3 ± 0.68 and 1.4 ± 0.78, respectively. The wettability of aminated nanofibers is attributed to the amine groups, in this sense the EGF incorporates many polar sites corresponding to the amino acids in EGF. These results are in agreement with other works which report values close to 0° [41,47,48]. The change of the contact angle with the time reveals a decrease in the value until a value of 0 in 5.56 s. The opposite trend is shown with contact surface area which increases its value near to 30 mm^2^ in 5.56 s. These results indicated that the aminated nanofibers with EGF resulting highly hydrophilic. This property is an important factor for the adhesion and attachment of cells and proteins. This property is an indicative of the possible biocompatibility of the material, this is in agreement with other works [47].

Additionally, an EGF release test without lipases and without amylases was carried out to confirm that the EFG is grafted on the surface of the nanofibers. The aminated nanofibers with EGF were immersed in phosphate buffer solution at pH of 7 and 0.1 M concentration, the absorbance was measured at 280 nm from 0 to 14 days. The results did not show an EGF release under experimental conditions confirming the EGF is grafted to surface.

### 3.2. In Vitro Biocompatibility Study (3T3 Cell Culture)

**Figure 6:**
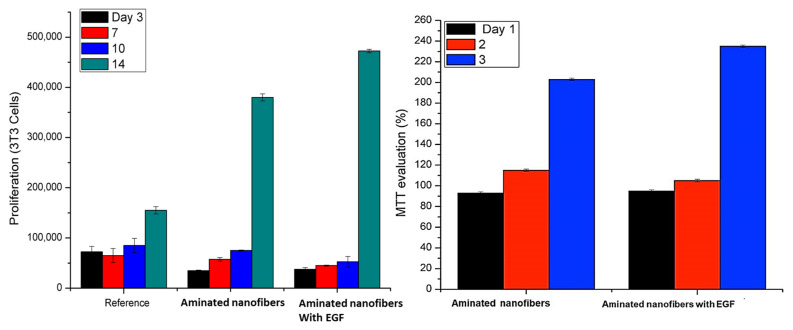
Mouse fibroblast (3T3) biocompatibility (**a**) and cell Proliferation MTT (3 days) (**b**) Cell counting (14 days).

The biocompatibility of the aminated nanofibers with EGF was evaluated and compared with aminated nanofibers by MTT method. In Figure 6, MTT evaluation and cell proliferation are shown, the cell proliferation in aminated nanofibers at one day was 90%, on the second day the proliferation increased, and on the third day the proliferation was close to 200%. These increments can be due to the amine groups that favor the cell compatibility [49,50,51]. However, the stimulus was not maintained for a long period of time in this case. The aminated nanofibers with EGF showed during the three days values close to 250% without reduction in the cell proliferation. The number of cells increased as result of an accelerated cell growth attributed to the high mitogenic activity of EGF. It is worthy to mention that the effective interaction with EGF receptors of adhered cells occurs when there is no steric hindrance. Additionally, when EGF is immobilized there is a high mitogenic activity that is persistent since it is believed that the immobilization of EGF inhibits internalization and decreases regulation [52]. Some works define a biocompatible material such as one that does not release any toxic matter and support the cells growth that can be evaluated by MTT assay [53].

The cell proliferation on the material was followed until day 14 with a constant increase of cell population. At the end of day 14, the cell population increased around four and five times, for aminated nanofibers and the aminated nanofibers with EGF material, respectively. These results agree with the proliferation step in the wound healing process. The increase in the cell population is attributed to the amine groups and EGF on the nanofibers surface improving the biocompatibility of the PCL.

**Figure 7:**
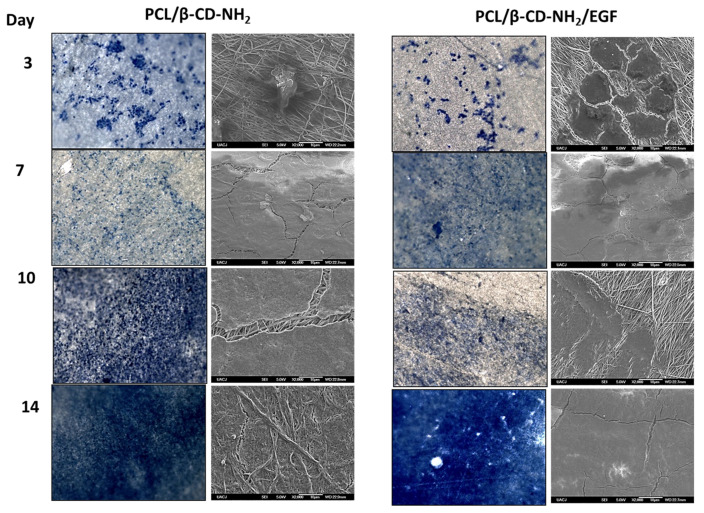
3T3 mouse cells seeded on scaffolds (aminated nanofibers and aminated nanofibers with EGF) analyzed on days 3, 7, 10, and 14; Methylene blue staining cells, images 40× (colored images on left). Scanning electron microscopy (SEM) micrographs 2000× (gray images to the right).

The obtained results suggest that the aminated nanofibers with and without EGF can present a good biocompatibility for wound healing due to the good adhesion, cell growth, and proliferation. The fibroblasts adhesion and proliferation on the nanofibers were followed by optical and electronic microscopy (SEM) during its culture assay as can be seen in Figure 7. A good cell adhesion was observed on the samples for both cases. Further, when the cells began the interaction with the nanofibers, evidencing cells colonies shaped by the interfaced nanofiber [54,55]. The results show increasing in surface covered by the epithelial cells over time. After 14 days of culture, the surface of nanofibers aminated with EGF were covered almost entirely by viable cells, in comparison with aminated nanofibers without EGF which shows less surface covered, considering the morphologic changes compared to the reference culture. These results were in agreement with the MTT results previously described (Figure 5), which revealed a significatively difference in cells proliferation on aminated nanofibers with EGF that contrast with the nanofibers without EGF. Additionally, the biocompatibility assay at 14 days was also in agreement with the SEM micrography showed a cell proliferation count around 450,000 cells for aminated nanofibers with EGF in comparison with 400,000 cells for aminated nanofibers. Both, the aminated nanofibers with and without EGF showed a good growth and cell adhesion. Moreover, it is assumed that the high level of wettability of both materials have a positive influence on the growth and cell adhesion [1,56]. The results show that it is possible to grow cells directly on scaffolds using fresh unmanipulated cells.

**Figure 8:**
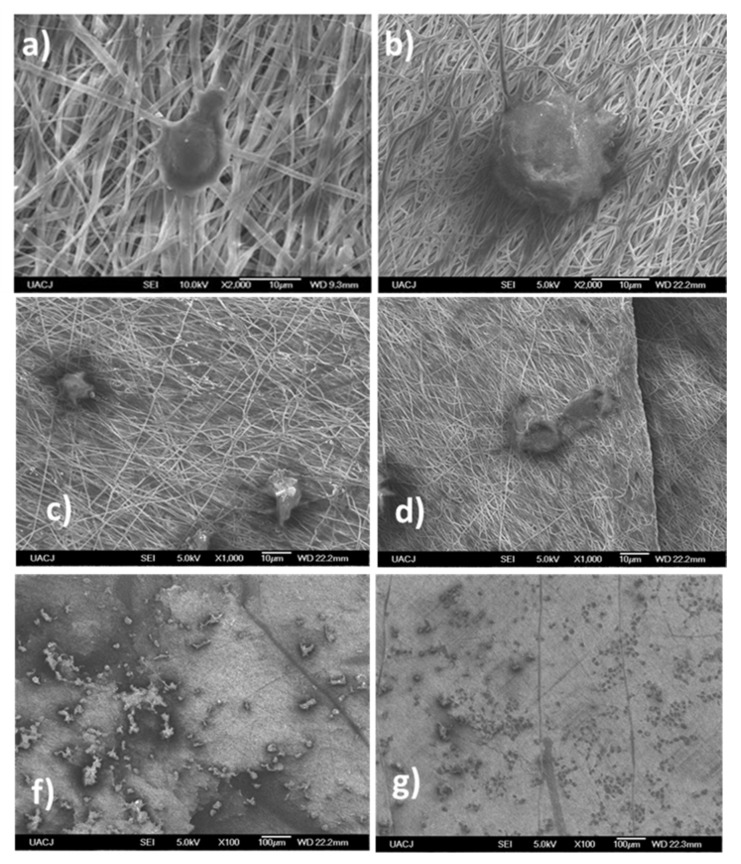
Evaluation of the integration and anchoring of 3T3 Mouse cells seeded on nanofibers on day 14 after wound at different magnifications for aminated nanofibers: (**a**) 100×, (**c**) 1000×, (**f**) 2000×; and aminated nanofibers with EGF: (**b**) 100×, (**d**) 1000×, (**g**) 2000×.

The morphology and in vitro cytocompatibility on aminated nanofibers and aminated nanofibers with EGF were evaluated by FESEM. The growth of 3T3 cell line on the nanofibrous mats during the culture period was continued and observed on surface of the nanofibers in agreement with previously reported for other electrospun scaffolds [57]. Moreover, the cells grow, interacted, and integrated effectively onto the nanofibers. The fiber orientation showed the 3T3 cells on aminated nanofibers without and with EGF showed a flatted shaped morphology. Figure 8 shows SEM micrographs of fibroblast cells cultured in final time on both fibrillar materials. The electrospun materials had a high cell density as well as a homogenous distribution, the fibroblast cells adhesion and spreading were improved, suggesting that the fibers are non-toxic for fibroblast cells. It can be inferred and confirmed that the presence of amino groups and EGF facilitate the growth of normal morphologies 3T3 cells on the nanofiber mats.

### 3.3. In Vivo Analysis

**Figure 9:**
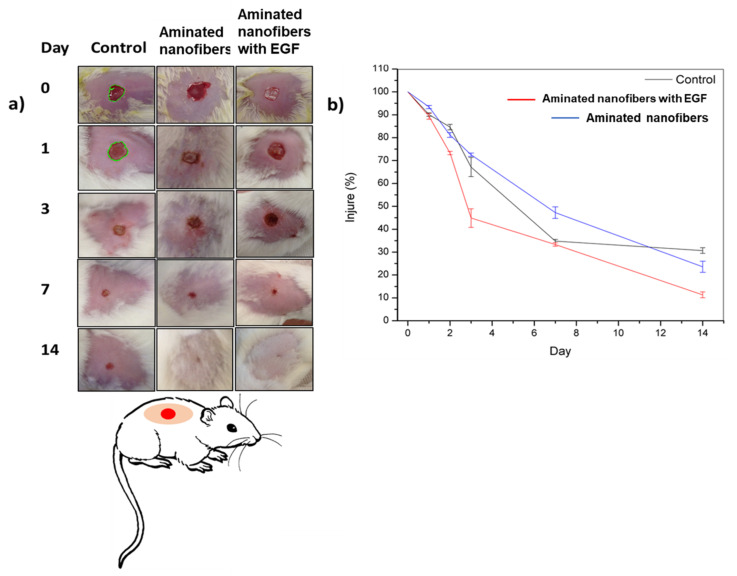
In vivo assay of tissue wound healing in rat model (**a**) wounds healing evolution; (**b**) graph wound healing percentage in the time (control: wounds without scaffold).

In order to evaluate these mats for wound healing, wounds of 1 cm in diameter were created by circular excision on the dermis and epidermis in the back of murine specimens. They were daily evaluated for 14 days and the analysis was made by triplicate. The observation periods were chosen according to the in vitro cell proliferation and cells population counts, using an untreated rat as control. Additionally, the wound healing was determined by wound area reduction and reported by healing percentage (Equation (2)), the results are shown on Figure 9. During the first three days an increase in the healing with the aminated nanofibers with EGF was observed (55%). Meanwhile, the control and aminated nanofibers material had similar healing (30%). On the seventh day, the aminated nanofibers with EGF and control murine had a similar healing (65%) in contrast with the aminated nanofibers with a 50% healing. Finally, the aminated nanofibers with EGF promoted 90% of healing, the aminated nanofibers presented 80% of healing and the control had 70% of healing, all of them by the day 14. Both materials showed a constant increase in the healing process during evaluation time. Even though the control did not show any significant changes between days 7 and 14. It must be highlighted that the nanofibers were absorbed, and that no adverse reaction was observed.

The in vivo results are in agreement with the in vitro study, which exhibited the best cell proliferation on the aminated nanofibers with EGF, followed by the aminated nanofibers without EGF. The murine accepted the aminated nanofibers with EGF without any complication, there was no loss of weight, neither macroscopic signs of inflammation or reddening of the tissue, the implant did not present fibrosis at all. The aminated nanofibers with EGF presented an accelerated healing compared with aminated nanofibers and the control.

## 4. Conclusions

Biomaterial for skin wound healing developed with aminated nanofibers with and without EGF were successfully prepared. The morphology did not change during neither after the grafted process, the number of EGF attached to the fibers were 2.67 × 10^14^ molecules per square centimeter. The in vitro assay showed that the aminated nanofibers and the aminated nanofibers with EGF improved their biocompatibility in comparison with only PCL nanofibers. In vivo tests showed that aminated nanofibers with EGF can accelerate the wound healing process in comparison with the others. This kind of biomaterials have the potential to be applied in wound healing, successfully.

## Data Availability

The data presented in this study are available on request from the corresponding author.
